# Supplementary material for: Functional dissection of the zDHHC palmitoyltransferase 5–golgin A7 palmitoylation complex
Source: J Biol Chem. 2025 Sep 8;301(10):110694. doi: 10.1016/j.jbc.2025.110694 (PMC12528901; doi:10.1016/j.jbc.2025.110694)
Supplement: Supporting Figure S1 [file mmc1.pdf]

A

Protein sequence conservation between mouse and human DHHC enzymes

Zdhhc	1	2	3	4	5	6	7	8	9	11	12	13	14	15	16	17	18	19	20	21	23	24	25
% identity	87	95	97	74	98	93	95	92	98	60	87	91	95	98	95	99	93	69	88	98	90	88	NA

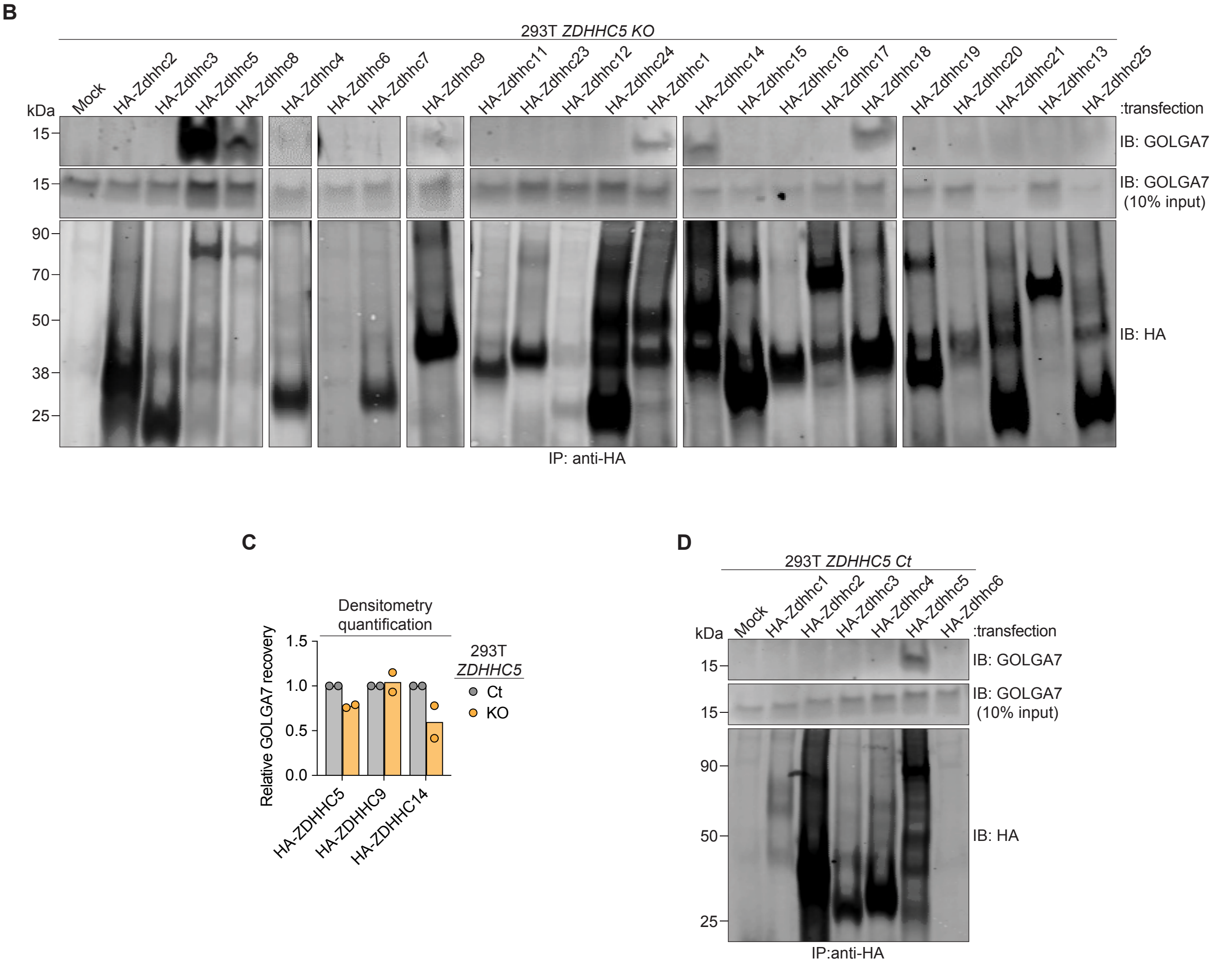

Figure S1. Interaction between Zdhhc-family proteins and endogenous GOLGA7, related to Figure 1. (A) Sequence conservation (% identity) between mouse Zdhhc enzymes and their human homologs. Mann-Whitney test, not significant (n.s.). (B) Immunoblot analysis from co-immunoprecipitation samples transfected as indicated. These blots are representative of two to three independent experiments and are from a large-scale screen. Due to the number of conditions, samples were transfected and processed in parallel across multiple experiments. Representative lanes were compiled from blots run and developed under identical conditions. Some samples are shown as intact blots, while others appear as single or double cropped lanes; this reflects differences in sample order and the presence of replicates on the same blot. Cropped lanes are delineated by white lines. All shown data derive from the same experimental series and were processed in parallel. Note: data for Zdhhc14-18 are also shown in Main Figure 1. (C) Densitometry quantification from two quantifiable replicates of relative GOLGA7 recovery, corresponding to Main Figure 1E, in *ZDHHC5* control (Ct) and knockout (KO) cell lines transfected as indicated. For each HA-ZDHHC construct, GOLGA7 recovery in the KO background is displayed relative to the corresponding Ct condition, which was normalized to 1.0. (D) Immunoblot analysis from co-immunoprecipitation samples transfected as indicated. Blot is representative of two independent experiments.
