# Supplementary material for: Functional dissection of the zDHHC palmitoyltransferase 5–golgin A7 palmitoylation complex
Source: J Biol Chem. 2025 Sep 8;301(10):110694. doi: 10.1016/j.jbc.2025.110694 (PMC12528901; doi:10.1016/j.jbc.2025.110694)
Supplement: Supporting Figure S2 [file mmc2.pdf]

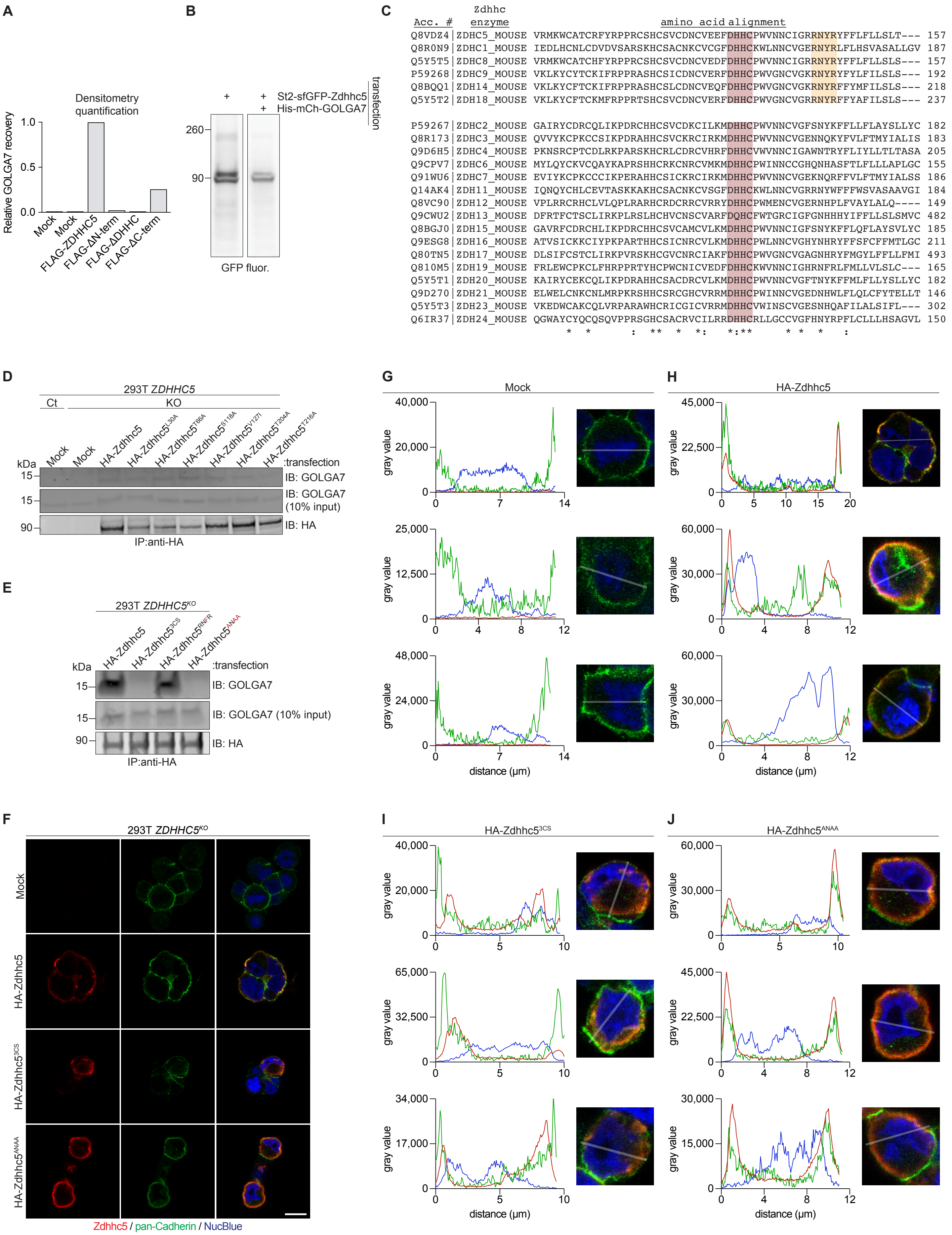
A
Densitometry quantification
Relative GOLGA7 recovery
1.0
0.5
0.0
Mock
Mock
FLAG-ZDHHC5
FLAG-ΔN-term
FLAG-ΔDHHC
FLAG-ΔC-term
B
St2-sfGFP-Zdhhc5
His-mCh-GOLGA7
transfection
260
90
GFP fluor.
C
Zdhhc
Acc. #
enzyme
amino acid alignment
Q8VDZ4|ZDHC5_MOUSE VRMKWCATCRFYRPPRCSHCSVCDNCVEEFDHHCPWVNNCIGRRNYRYFFLFLLSLT--- 157
Q8R0N9|ZDHC1_MOUSE IEDLHCNLCDVDVSARSKHCSACNKCVCGFDHHCKWLNNCVGERNYRLFLHSVASALLGV 187
Q5Y5T5|ZDHC8_MOUSE VRMKWCATCHFYRPPRCSHCSVCDNCVEDFDHHCPWVNNCIGRRNYRYFFLFLLSLS--- 157
P59268|ZDHC9_MOUSE VKLKYCYTCKIFRPPRASHCSICDNCVERFDHHCPWVGNCVGKRNYRYFYLFILSLS--- 192
Q8BQQ1|ZDH14_MOUSE VKLKYCFTCKIFRPPRASHCSLCDNCVEQFDHHCPWVGNCVGKRNYRFFYMFILSLS--- 218
Q5Y5T2|ZDH18_MOUSE VKLKYCFTCKMFRPPRTSHCSVCDNCVERFDHHCPWVGNCVGRRNYRFFYAFILSLS--- 237
P59267|ZDHC2_MOUSE GAIRYCDRCQLIKPDRCHHCSVCDKCILKMDHHCPWVNNCVGFSNYKFFLLFLAYSLLYC 182
Q8R173|ZDHC3_MOUSE QVVYKCPKCCSIKPDRAHHCSVCKRCIRKMDHHCPWVNNCVGENNQKYFVLFTMYIALIS 183
Q9D6H5|ZDHC4_MOUSE PKNSRCPTCDLRKPARSKHCRLCDRCVHRFDHHCVWVNNCIGAWNTRYFLIYLLTLTASA 205
Q9CPV7|ZDHC6_MOUSE MYLQYCKVCQAYKAPRSHHCRKCNRCVMKMDHHCPWINNCCGHQNHASFTLFLLLAPLGC 155
Q91WU6|ZDHC7_MOUSE EVIYKCPKCCCIKPERAHHCSICKRCIRKMDHHCPWVNNCVGEKNQRFFVLFTMYIALSS 186
Q14AK4|ZDH11_MOUSE IQNQYCHLCEVTASKKAKHCSACNKCVSGFDHHCKWLNNCVGRRNYWFFFWSVASAAVGI 184
Q8VC90|ZDH12_MOUSE VPLRRCRHCLVLQPLRARHCRDCRRCVRRYDHHCPWMENCVGERNHPLFVAYLALQ---- 149
Q9CWU2|ZDH13_MOUSE DFRTFCTSCLIRKPLRSLHCHVCNSCVARFDQHCFWTGRCIGFGNHHHYIFFLLSLSMVC 482
Q8BGJ0|ZDH15_MOUSE GAVRFCDRCHLIKPDRCHHCSVCAMCVLKMDHHCPWVNNCIGFSNYKFFLQFLAYSVLYC 185
Q9ESG8|ZDH16_MOUSE ATVSICKKCIYPKPARTHHCSICNRCVLKMDHHCPWLNNCVGHYNHRYFFSFCFFMTLGC 211
Q80TN5|ZDH17_MOUSE DLSIFCSTCLIRKPVRSKHCGVCNRCIAKFDHHCPWVGNCVGAGNHRYFMGYLFFLLFMI 493
Q810M5|ZDH19_MOUSE FRLEWCPKCLFHRPPRTYHCPWCNICVEDFDHHCKWVNNCIGHRNFRLFMLLVLSLC--- 165
Q5Y5T1|ZDH20_MOUSE KAIRYCEKCQLIKPDRAHHCSACDRCVLKMDHHCPWVNNCVGFTNYKFFMLFLLYSLLYC 182
Q9D270|ZDH21_MOUSE ELWELCNKCNLMRPKRSHHCSRCGHCVRRMDHHCPWINNCVGEDNHWLFLQLCFYTELLT 146
Q5Y5T3|ZDH23_MOUSE VKEDWCAKCQLVRPARAWHCRICGICVRRMDHHCVWINSCVGESNHQAFILALSIFL--- 302
Q6IR37|ZDH24_MOUSE QGWAYCYQCQSQVPPRSGHCSACRVCILRRDHHCRLLGCCVGFHNYRPFLCLLLHSAGVL 150
* * : ** * *: *:** * * * :
D
293T ZDHHC5
Ct
KO
Mock
Mock
HA-Zdhhc5
HA-Zdhhc5 L30A
HA-Zdhhc5 T66A
HA-Zdhhc5 S118A
HA-Zdhhc5 V127I
HA-Zdhhc5 T204A
HA-Zdhhc5 T216A
:transfection
kDa
15
15
90
IB: GOLGA7
IB: GOLGA7 (10% input)
IB: HA
IP:anti-HA
E
293T ZDHHC5 KO
HA-Zdhhc5
HA-Zdhhc5 3CS
HA-Zdhhc5 RNFR
HA-Zdhhc5 ANAA
:transfection
kDa
15
15
90
IB: GOLGA7
IB: GOLGA7 (10% input)
IB: HA
IP:anti-HA
F
293T ZDHHC5 KO
Mock
HA-Zdhhc5
HA-Zdhhc5 3CS
HA-Zdhhc5 ANAA
Zdhhc5 / pan-Cadherin / NucBlue
G
Mock
40,000
20,000
0
25,000
12,500
0
48,000
24,000
0
gray value
0 7 14
0 4 8 12
0 7 14
distance (µm)
H
HA-Zdhhc5
45,000
22,500
0
60,000
30,000
0
60,000
30,000
0
gray value
0 5 10 15 20
0 4 8 12
0 4 8 12
distance (µm)
I
HA-Zdhhc5 3CS
40,000
20,000
0
65,000
32,500
0
34,000
17,000
0
gray value
0 5 10
distance (µm)
J
HA-Zdhhc5 ANAA
60,000
30,000
0
45,000
22,500
0
30,000
15,000
0
gray value
0 5 10
0 4 8 12
distance (µm)

Figure S2. Interaction between Zdhhc5 mutants and endogenous GOLGA7, related to Figure 2. (A) Densitometry quantification corresponding to Main Figure 2B, transfected as indicated. Values less than or equal to 0 were set to 0.01 for visibility; actual quantification yielded no detectable band above background. (B) GFP fluorescence of lysate from 293T cells transfected as indicated. Lanes shown side-by-side were cropped from the same blot and are shown adjacent for clarity. They are representative of two independent experiments. (C) Clustal Omega amino acid alignment of the Zdhhc mouse family conserved cysteine rich domain and DHHC active site (red), highlighting the RNYR motif in conserved GOLGA7 binders (top) vs. nonbinders (bottom). (D-E) Immunoblot analysis of co-immmunoprecipitation samples in Control (Ct) and *Zdhhc5* gene disrupted ("KO") cells transfected as indicated. Representative of two independent experiments. (F) Immunofluorescence of 293T *Zdhhc5*^{KO} cells transfected as indicated. Scale bar = 10 µm. (G-J) Fluorescence intensity line profile analysis of cells corresponding to (F) and transfected as indicated. Three cells were quantified per condition. (D-J) Red=Zdhhc5, Green=pan-Cadherin, Blue=NucBlue. All images are representative of two independent experiments.
