# Supplementary material for: Functional dissection of the zDHHC palmitoyltransferase 5–golgin A7 palmitoylation complex
Source: J Biol Chem. 2025 Sep 8;301(10):110694. doi: 10.1016/j.jbc.2025.110694 (PMC12528901; doi:10.1016/j.jbc.2025.110694)
Supplement: Supporting Figure S3 [file mmc3.pdf]

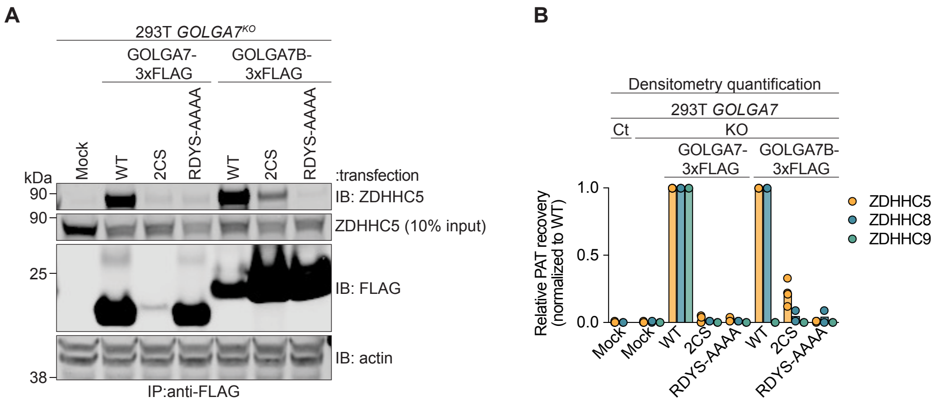

Figure S3. ZDHHC5 interaction with GOLGA7 and GOLGA7B mutants, Related to Figure 3. (A) Co-immunoprecipitation analysis of ZDHHC5 binding in *GOLGA7* gene disrupted (”KO”) cell lines transfected as indicated. The GOLGA7$^{RDYS\text{-}AAAA}$ mutant is shown in a high-expression replicate. (B) Densitometry quantification corresponding to (A) and Main Figure 3G, of endogenous PAT recovery in 293T *GOLGA7* control (Ct) and KO cells transfected with the indicated GOLGA7- and GOLGA7b-3xFLAG constructs. Recovery was normalized to total 3xFLAG-tagged bait protein in each lane and displayed relative to the corresponding WT construct, which was set to 1.0. Values less than or equal to 0 (i.e., no detectable band above background) were set to 0.01 for visibility. Quantification of ZDHHC9 recovery was only possible for the GOLGA7-3xFLAG “WT” condition; all other constructs yielded no detectable ZDHHC9 signal. Data represent four independent replicates.
