# Supplementary material for: Functional dissection of the zDHHC palmitoyltransferase 5–golgin A7 palmitoylation complex
Source: J Biol Chem. 2025 Sep 8;301(10):110694. doi: 10.1016/j.jbc.2025.110694 (PMC12528901; doi:10.1016/j.jbc.2025.110694)
Supplement: Supporting Figure S4 [file mmc4.pdf]

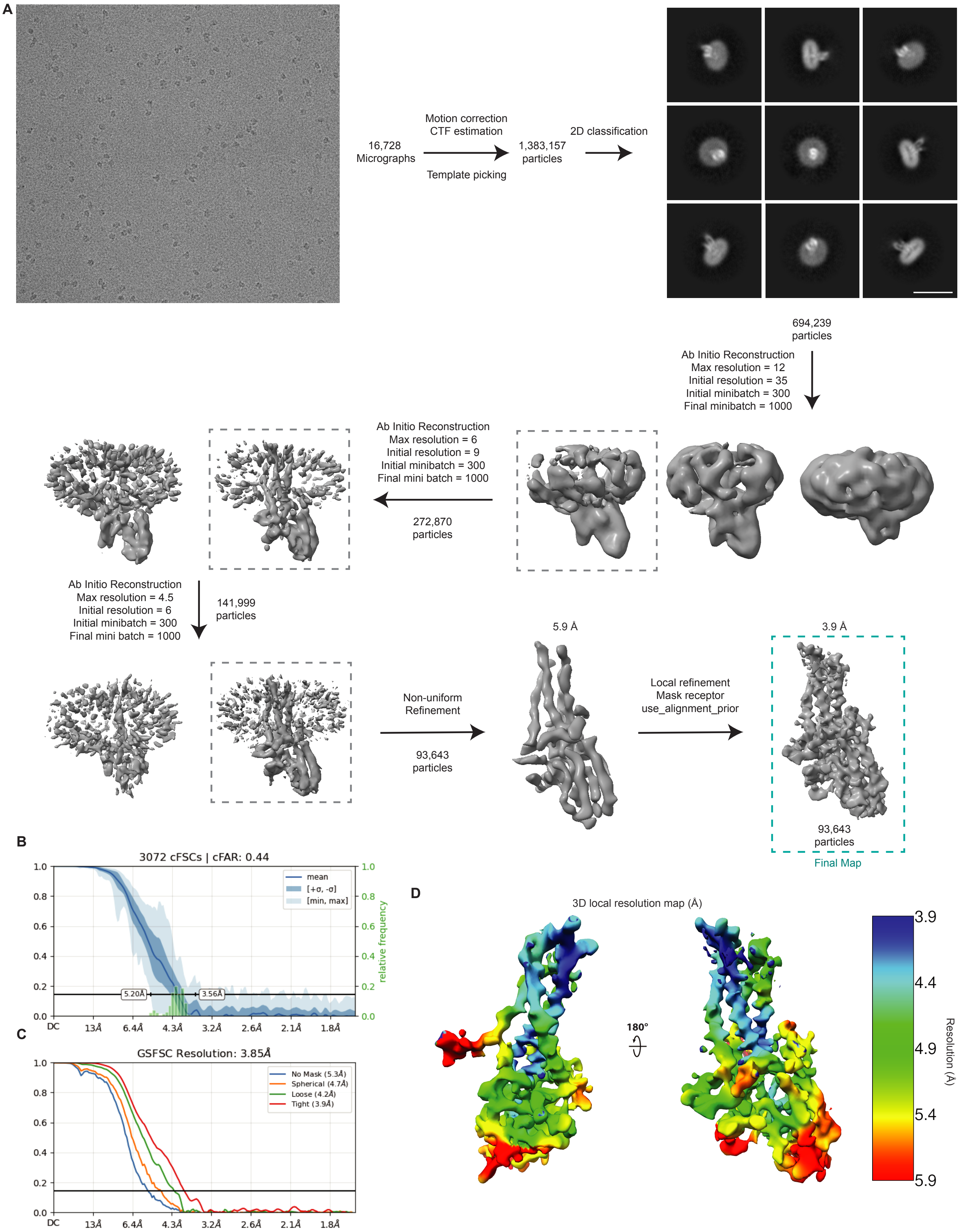
A
16,728
Micrographs
Motion correction
CTF estimation
Template picking
1,383,157
particles
2D classification
694,239
particles
Ab Initio Reconstruction
Max resolution = 12
Initial resolution = 35
Initial minibatch = 300
Final minibatch = 1000
Ab Initio Reconstruction
Max resolution = 6
Initial resolution = 9
Initial minibatch = 300
Final mini batch = 1000
272,870
particles
Ab Initio Reconstruction
Max resolution = 4.5
Initial resolution = 6
Initial minibatch = 300
Final mini batch = 1000
141,999
particles
Non-uniform
Refinement
93,643
particles
5.9 Å
Local refinement
Mask receptor
use_alignment_prior
3.9 Å
93,643
particles
Final Map
B
3072 cFSCs | cFAR: 0.44
mean
[+σ, -σ]
[min, max]
relative frequency
5.20Å
3.56Å
DC
13Å
6.4Å
4.3Å
3.2Å
2.6Å
2.1Å
1.8Å
C
GSFSC Resolution: 3.85Å
No Mask (5.3Å)
Spherical (4.7Å)
Loose (4.2Å)
Tight (3.9Å)
D
3D local resolution map (Å)
180°
3.9
4.4
4.9
5.4
5.9
Resolution (Å)

Figure S4. Cryo-EM data analysis of the Zdhhc5-GOLGA7 complex, related to Figure 4. (A) A representative cryo-micrograph from the chosen grid and flowchart of EM data processing including representative two-dimensional class averages (scale bar = 130 Å). (B) Directional corrected FSC (cFSC) curves from CryoSPARC showing resolution anisotropy across 3D orientations. A cFAR value of 0.44 indicates moderate anisotropy. Relative frequency (right y-axis) reflects orientation sampling density. (C) Gold standard Fourier shell correlation (GSFSC) curves for final 3D reconstruction. FSC curves were calculated in cryoSPARC using different masking conditions: no mask (blue), spherical mask (orange), loose mask (green), and tight mask (red). The reported resolution (3.85 Å) is defined at the 0.143 FSC threshold. (D) Local resolution estimation of the final cryo-EM map. Two views of the 3D reconstruction are shown colored by local resolution as estimated by CryoSPARC, with resolution ranging from 3.9 Å (blue) to 5.9 Å (red).
