# Supplementary material for: Functional dissection of the zDHHC palmitoyltransferase 5–golgin A7 palmitoylation complex
Source: J Biol Chem. 2025 Sep 8;301(10):110694. doi: 10.1016/j.jbc.2025.110694 (PMC12528901; doi:10.1016/j.jbc.2025.110694)
Supplement: Supporting Figure S5 [file mmc5.pdf]

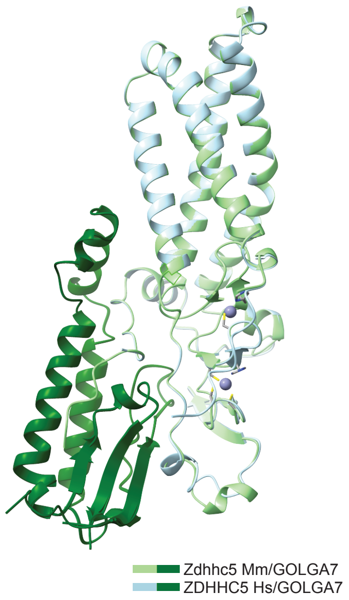

Figure S5. AlphaFold3 models of human and mouse ZDHHC5/GOLGA7 complexes, related to Figure 5. AlphaFold3 generated model of ZDHHC5 Hs and Zdhhc5 Mm in complex with GOLGA7 Hs, generated using the following UniProt accessions: Q9C0B5 (Hs ZDHHC5), Q8VDZ4 (Mm Zdhhc5), Q7Z5G4 (Hs GOLGA7).
