# Supplementary material for: Functional dissection of the zDHHC palmitoyltransferase 5–golgin A7 palmitoylation complex
Source: J Biol Chem. 2025 Sep 8;301(10):110694. doi: 10.1016/j.jbc.2025.110694 (PMC12528901; doi:10.1016/j.jbc.2025.110694)
Supplement: Supporting Figure S6 [file mmc6.pdf]

```
Protein                    Amino acid alignment
ZDHHC9  23   FCCDGRVMMARQKGIFYLTLFL-ILGTCTLFFAFECRYLAVQLS--PAIPVFAAMLFLFS  79
             F   GR    +     +L + L I+    LF  FE   L    +    + +F    ++ +
Erf2    61   FFLGGRFRTVKGAKPLWLGVLLAIVCPMVLFSIFEAHKLWHTQNGYKVLVIFFYYFWVIT  120

ZDHHC9  80   MATLLRTSFSDPGVIPRALPDEAAFIEMEIEATNGAVPQGQRPPPRIKNFQINNQIVKLK  139
             +A+ +RT+ SDPGV+PR        I +     N  +PQ       +      ++ + +K
Erf2    121  LASFIRTATSDPGVLPRN-------IHLSQLRNNYQIPQEYYNLITLPTHSSISKDITIK  173

ZDHHC9  140  YCYTCKIFRPPRASHCSICDNCVERFDHHCPWVGNCVGKRNYRYFYLFILSLSLLTIYVF  199
             YC +C+I+RPPR+SHCS C+ CV   DHHC WV NC+GKRNYR+F +F+L   L ++ +
Erf2    174  YCPSCRIWRPPRSSHCSTCNVCVMVHDHHCIWVNNCIGKRNYRFFLIFLLGAILSSVILL  233

ZDHHC9  200  AFNIVYVALKSLKIGFLETLKETPGTVLEVLICFFTLWSVVGLTGFHTFLVALNQTTNED  259
                 +++A +S         ++ P  +L +     TLW    L  +H F+    QTT E
Erf2    234  TNCAIHIARES------GGPRDCPVAILLLCYAGLTLWYPAILFTYHIFMAGNQQTTREF  287

ZDHHC9  260  IKGSWTGKNRV-------QNPYSHGNIVKNCCEVLCGPLPPSVLDRR  299
             +KG  + KN V       +N Y+ G+ +KN   ++  P  PS +  R
Erf2    288  LKGIGSKKNPVFHRVVKEENIYNKGSFLKNMGHLMLEPRGPSFVSAR  334
```

Figure S6. Amino acid alignment, related to Figure 5. Clustal Omega amino acid alignment of human ZDHHC9 and yeast ERF2. Red highlights the conserved DHHC active site; orange highlights the RNYR site conserved among GOLGA7 binders.
