# Supplementary material for: Functional dissection of the zDHHC palmitoyltransferase 5–golgin A7 palmitoylation complex
Source: J Biol Chem. 2025 Sep 8;301(10):110694. doi: 10.1016/j.jbc.2025.110694 (PMC12528901; doi:10.1016/j.jbc.2025.110694)
Supplement: Supporting Table S1 [file mmc7.docx]

|  | Zdhhc5-GOLGA7 |
| --- | --- |
| **Data deposition** |  |
| PDB | 9OA6 |
| EMDB | 70274 |
|  |  |
| **Cryo-EM Data Collection** |  |
| Voltage (kV) | 300 |
| Magnification (×) | 105,000 |
| Pixel size (Å) | 0.832 |
| Electron exposure (e^-^/Å^2^) | 80 |
| Defocus range (μm) | -0.8 to -2.0 |
| Microscope | Titan Krios |
| Camera | Gatan K3 |
|  |  |
| **Cryo-EM Data Processing** |  |
| Initial number of particles | 1,383,157 |
| Final number of particles | 93,643 |
| Symmetry imposed | C1 |
| Map resolution (Å) | 3.9 |
| FSC threshold | 0.143 |
| Map resolution range (Å) | 3.7-5.8 |
|  |  |
| **Model Refinement** |  |
| Reference model used | AF3-predicted structure |
| Model resolution (Å) | 4.0 |
| FSC threshold | 0.143 |
| Refinement package | Phenix real-space refinement |
| Model composition |  |
| Chains | 2 |
| Non-hydrogen atoms | 2909 |
| Protein residues | 369 |
| Ligands | 2 Zn^2+^ |
|  |  |
| **Model Validation** |  |
| B factors (Å²) |  |
| Protein | 49.1-178.4 (mean: 103.2) |
| Ligand | 181.4-190.9 (mean 186.15) |
| R.m.s. deviations |  |
| Bond lengths (Å) | 0.003 |
| Bond angles (°) | 0.784 |
| Validation |  |
| MolProbity score | 2.19 |
| Clash score | 11.94 |
| Rotamer outliers (%) | 0.0 |
| Cβ outliers (%) | NA |
| Ramachandran plot (%) |  |
| Favored | 87.95 |
| Allowed | 11.78 |
| Outliers | 0.27 |

**Supplementary Table 1, related to Figure 4.** Summary statistics for the cryo-EM model of Zdhhc5-GOLGA7.
